# In-hospital management of children with bacterial meningitis in Italy

**DOI:** 10.1186/s13052-014-0087-1

**Published:** 2014-11-14

**Authors:** Marta Ciofi degli Atti, Susanna Esposito, Luciana Parola, Lucilla Ravà, Gianluigi Gargantini, Riccardo Longhi, the GSAQ working group

**Affiliations:** Unit of Clinical Epidemiology, Bambino Gesù Children’s Hospital, Rome, Italy; Pediatric Highly Intensive Care Unit, Department of Pathophysiology and Transplantation, Università degli Studi di Milano, Fondazione IRCCS Ca’ Granda Ospedale Maggiore Policlinico, Milan, Italy; Department of Pediatrics, AO Ospedale Civile di Legnano, G.Fornaroli Hospital, Magenta, Milan, Italy; Department of Pediatrics, Lodi Hospital, Lodi, Milan, Italy; Department of Pediatrics, AO Sant’Anna, Como, Italy

**Keywords:** Bacterial meningitis, Children, Meningitis sequelae, *Neisseria meningitidis*, *Streptococcus pneumoniae*

## Abstract

**Background:**

Over the years 2009–2013, we conducted a prospective study within a network established by the Italian Society of Pediatrics to describe the in-hospital management of children hospitalized for acute bacterial meningitis in 19 Italian hospitals with pediatric wards.

**Methods:**

Hospital adherence to the study was voluntary; data were derived from clinical records. Information included demographic data, dates of onset of first symptoms, hospitalization and discharge; diagnostic evaluation; etiology; antimicrobial treatment; treatment with dexamethasone; in-hospital complications; neurological sequelae and status at hospital discharge. Characteristics of in-hospital management of patients were described by causative agent.

**Results:**

Eighty-five patients were identified; 49.4% had received an antimicrobial treatment prior to admission. Forty percent of patients were transferred from other Centers; the indication to seek for hospital care was given by the primary care pediatrician in 80% of other children. Etiological agent was confirmed in 65.9% of cases; the most common infectious organism was *Neisseria meningitidis* (34.1%), followed by *Streptococcus pneumoniae* (20%). Patients with pneumococcal meningitis had a significant longer interval between onset of first symptoms and hospital admission. Median interval between the physician suspicion of meningitis and in-hospital first antimicrobial dose was 1 hour (interquartile range [IQR]: 1–2 hours). Corticosteroids were given to 63.5% of cases independently of etiology; 63.0% of treated patients received dexamethasone within 1 hour of antibiotic treatment, and 41.2% were treated for ≤4 days. Twenty-nine patients reported at least one in-hospital complication (34.1%). Six patients had neurological sequelae at discharge (7.1%). No deaths were observed.

**Conclusions:**

We observed a rate of meningitis sequelae at discharge similar to that reported by other western countries. Timely assistance and early treatment could have contributed to the favorable outcome that was observed in the majority of cases. Adherence to recommendation for corticosteroid adjunctive therapy seems suboptimal, and should be investigated in further studies. Most meningitis cases were due to *N. meningitidis* and *S. pneumoniae*. Reaching and maintaining adequate vaccination coverage against pneumococcal and meningococcal invasive infections remains a priority to prevent bacterial meningitis cases.

## Background

In the last 20 years, the availability of protein conjugate vaccines against *Haemophilus influenzae* type b (Hib), *Streptococcus pneumoniae*, and *Neisseria meningitidis* has deeply modified the epidemiology of bacterial meningitis, with a dramatic reduction in incidence documented in Countries with universal vaccination programs [1-3]. In Italy, Hib vaccination was recommended since early 1990s, and coverage rates >95% have been achieved [4]. Meningococcal and pneumococcal conjugate vaccines have been introduced in early 2000’s, with estimated vaccination coverage rates <70% [5]. In Italy, national surveillance of bacterial meningitis was established in 1994; in 2004, national surveillance was extended to all invasive diseases due to Hib, *S. pneumoniae*, and *N. meningitidis*. Over the years 2007–2012, a mean of 130 meningitis due to *N. meningitidis* and *S. pneumoniae* have been reported annually in children aged 0–14 years, while less than 10 cases of Hib meningitis per year were reported in the same age group [6]. National surveillance data do not provide information on in-hospital management of cases.

Early recognition of signs and symptoms suggestive of bacterial meningitis, along with prompt administration of empirical antimicrobial treatment are crucial to improve clinical outcome [7,8]. Timely diagnostic evaluation is also paramount to confirm etiology and modify antimicrobial treatment for optimal therapy. Guidelines for the management of bacterial meningitis in adults and children have been published [9,10]; however, various studies have documented criticalities in implementation of recommendations regarding diagnostic evaluation, antimicrobial and supportive treatment [11-14]. In 2008, the Study Group for Accreditation and Quality Improvement of the Italian Society of Pediatric launched a network for monitoring clinical management of children hospitalized for acute bacterial meningitis. In this article, we presented the results of the data prospectively collected by 19 Italian hospitals, from January 2009 to June 2013.

## Methods

The objective of this study was to evaluate diagnostic methods and treatment of children aged 1 month to 18 years of age discharged with diagnosis of bacterial meningitis. Nineteen hospitals voluntary participated to the study; these included two tertiary care teaching children’s hospitals (in northern and central Italy, respectively), three pediatric departments of tertiary-care teaching hospitals (two in northern Italy, one in southern Italy), and fourteen pediatric units of other hospitals (all in northern Italy). Bacterial meningitis was defined according to physician’s clinical diagnosis at discharge, including either laboratory-confirmed or probable cases, i.e., patients with compatible signs and symptoms where cerebrospinal fluid (CSF) examination showed turbid appearance; and/or leukocytosis (>100 cells/mm^3^). Laboratory confirmation included identification of bacteria directly (by culture or polymerase chain reaction [PCR] from CSF or blood), or indirectly (by antigen detection on CSF).

This study was approved by the Ethics Committee of all the participating Centers, in compliance with the Helsinki Declaration. Written informed consent was obtained from all the parents and participants aged ≥8 years gave their written assent. Information derived from clinical records included demographic data (gender, age), dates of onset of first symptoms, hospitalization and discharge; diagnostic evaluation (lumbar puncture at hospital admission, CSF formula, Gram stain, culture, latex agglutination and PCR; blood culture and PCR); etiology (confirmation of bacterial pathogen; availability of in vitro susceptibility testing within 3 days since hospital admission); antimicrobial treatment (treatment prior to admission; timing of in-hospital first dose administration, defined as interval between suspected diagnosis of meningitis and first in-hospital antibiotic administration; agents used for treatment); treatment with dexamethasone (administration, timing and duration); imaging (cranial computed tomography [CT]; cranial magnetic resonance imaging [MRI]; transfontanellar ultrasound); audiological assessment; in-hospital complications; neurological sequelae and status at hospital discharge.

Data were entered by participating hospitals in a web-based database, protected in terms of security.

To ensure completeness of data collection, hospitals reported monthly by e-mail if they did not observe cases.

All data were anonymized and exported to STATA 13.1 software (Stata Corp., College Station, TX, USA) for analysis. The database was analyzed for verifying and excluding duplicates. Proportions were calculated excluding missing values. Characteristics of hospital management of patients were described by causative agent (*N. meningitidis*, *S. pneumoniae*, other agents). Comparisons between groups were made with Chi-square or Fisher exact tests for comparing proportions of categorical variables, and with Kruskall Wallis test for comparing medians of continuous variables.

## Results

### Patient characteristics and diagnostic evaluations

Eighty-five patients were identified; the number of patients reported by hospital ranged from 1 to 25, with 72.9% of total cases (62/85) admitted to four tertiary care hospitals. Patient characteristics are shown in Table 1.

**Table 1:** **Characteristics of patients and diagnostic evaluation at hospital admission**

		**Total = 85**	
	**N**	**%**	**95** **%** **CI**
**Age**			
1-11 months	2	2.4	0.2 – 8.2
1-4 years	27	31.8	22.1 – 42.6
5-9 years	24	28.2	19.0 – 39.0
10-14 years	27	31.8	22.1 – 42.6
15-18 years	5	5.9	1.9 – 13.2
**Gender**			
Male	55	66.3	53.5 – 74.8
Female	28	33.7	23.1 – 44.0
**Decision to seek hospital care**			
Parents	10	11.8	5.8 – 20.6
Transfer from other hospital	34	40.0	29.5 – 51.2
Primare care physician	41	48.3	37.3 – 59.3
**Diagnostic evaluation**			
Lumbar puncture at hospital admission	80	91.7	86.8 – 98.1
CSF culture	83	97.7	91.8 – 99.7
CSF antigens	56	65.9	54.8 – 75.8
CSF PCR	34	40.0	29.5 – 51.2
Blood culture	78	91.8	83.8 – 96.6
Blood PCR	28	32.9	23.1 – 44.0
**Known causative agent**	56	65.9	54.8 – 75.8
**In vitro susceptibility testing available < days since admission** (excluding unknown agent)	42	75.0	38.4 – 60.5

95% CI: 95% confidence interval; CSF: cerebrospinal fluid; PCR: polymerase chain reaction.

Forty percent of patients were transferred from other hospitals.

For patients who were not transferred from other centers, the indication to seek for hospital care was given by the primary care pediatrician in 80% of cases (41/51).

CSF culture was performed in all patients except for two, in whom blood cultures were done. Etiological agent was confirmed in 68.2% of cases; patients without microbiological confirmation had a median CSF white blood cell count (WBC) of 530/mmc (interquartile range [IQR]: 108–4.000 WBC/mmc), with a median polymorphonuclear leukocytes (PMNs) proportion of 70% (IQR: 60 - 82% PMNs) (data not shown in the table).

Interestingly, positivity rate for bacteria did not differ in patients in whom PCR testing was performed on CSF or blood (27/44; 61.3%), compared to patients in whom only culture and no PCR testing was done (29/41; 70.7%).

### Etiology and treatment

A 7-month old infant had a nosocomial infection due to *Enterococcus faecium*; he was treated with teicoplanin and aminoglycosides, and had no complications or sequelae at discharge.

All the other cases were community-acquired, with onset peaking in months from October to March (54/85; 63.5%).

The most common infectious organism was *N. meningitidis*, accounting for 34.1% of total cases (29 patients), followed by *S. pneumoniae* (20%; 17 patients), group A *Streptococcus* and *Streptococcus pyogenes* (5.9%; 5 patients), and *Staphylococcus* species (2.4%; 2 patients) (Table 2). Hib and *Mycoplasma pneumoniae* were identified in one patient each.

**Table 2:** **Characteristics of patients at hospital admission and treatment by causative agent**

	***N. meningitidis***	***S. pneumoniae***	**Other**	**Not known**	**p-value**	**Total**
	**N. (29)**	**N. (17)**	**N. (10)**	**N. (29)**		**N. (85)**
Median age at onset yrs – (IQR)	4.0 (1.5 – 7.6)	1.4 (0.5 - 4.6)	0.6 (0.3 - 7.0)	5.2 (0.4 – 8.2)	0.085	3.3 (0.5 - 7.1)
Median interval days between onset and hospital admission – (IQR)	1 (1–2)	3 (2–3)	1 (0–2)	1 (1–4)	0.005	1 (1–3)
Number of patients treated with antibiotics prior to hospitalization – (%)	14 (48.3)	8 (47.1)	2 (20.0)	18 (62.1)	0.238	42 (49.4)
Median interval hours between suspected diagnosis of meningitis and first in-hospital antibiotic administration – (IQR)	1 (1–2)	1 (1–2)	1 (1–2)	1 (1–2)	0.995	1 (1–2)
In vitro susceptibility testing available <3 days since admission, excluded unknown agent – (%)	22 (75.9)	12 (70.6)	8 (80.0)	-	0.691	42 (75.0)
Type of antibiotic administered – (%)					<0.001	
III-generation cephalosporin	18 (62.1)	2 (11.8)	1 (10.0)	16 (55.2)		29 (34.1)
III-generation cephalosporin + glycopeptides	3 (10.3)	7 (41.2)	1 (10.0)	1 (3.4)		17 (20.0)
III-generation cephalosporin + other antibiotics	8 (27.6)	8 (47.1)	5 (50.0)	11 (37.9)		10 (11.8)
Other	0 (0.0)	0 (0.0)	3 (30.0)	1 (3.4)		29 (34.1)
Number of patients treated with dexamethasone – (%)	15 (51.7)	14 (82.4)	6 (60.0)	19 (65.5)	0.222	54 (63.5)
Number of patients who received dexamethasone within 1 hour of antibiotic – (%)	9 (31.0)	10 (58.8)	5 (50)	10 (34.5)	0.544	34 (40)
Median duration days of dexamethasone treatment – (IQR)	6 (4–12)	4 (4–12)	7 (4–11)	6 (4–10)	0.951	6 (4 – 12)

IQR: interquartile range.

Characteristics of patients at hospital admission and antibiotic administered by causative agent are shown in Table 2. Median interval between onset of first symptoms and hospital admission did significantly vary by causative agent; patients with meningitis due to *S. pneumoniae* reported a median interval of 3 days, compared to a median interval of 1 day for all other groups.

Antibiotic was given prior to admission in participating hospitals to 42 patients overall (49.4%), with no differences by agent.

Median interval between the physician suspicion of meningitis and in-hospital first antimicrobial dose was 1 hour (IQR: 1–2 hours), independently of etiology. *In vitro* susceptibility testing results were available within 3 days since hospital admission for 75% of the patients with known etiological agent; in the remaining 25% of the patients, *in vitro* susceptibility testing were available after 4–6 days since CSF availability.

Type of antibiotic used for treatment did differ by causative agent. Third-generation cephalosporins (ceftriaxone or cefotaxime) were the only antimicrobial treatment administered to the majority of patients with confirmed *N. meningitidis* (62.1%) or without etiological confirmation (55.2%) (Table 2). Most patients with *S. pneumoniae* meningitis were treated with third-generation cephalosporins associated with glycopeptides (vancomicin or teicoplanin; 41.2%), or other antibiotics (47.1%).

Corticosteroids were given to 63.5% (54/85) of cases according to hospital’s protocol; the proportion of children treated with dexamethasone did not significantly vary by group (Table 2). When considering timing and duration of corticosteroid administration, 63.0% (34/54) of treated patients received dexamethasone within 1 hour of antibiotic treatment, and 41.2% (14/34) were treated for ≤4 days.

### Other investigations, complications and outcome at discharge

Imaging (CT scan, MRI or transfontanellar ultrasound) was performed by 64.7% (55/85) of patients; 48.2% underwent audiological evaluation prior to discharge, and a follow up audiological assessment was planned in 42.2% (Table 3). Twenty-nine patients reported at least one in-hospital complications, including 6 seizures, 5 disseminated intravascular coagulation (DIC) cases, 3 cases with septic shock, 1 cerebral hemorrhages, and 1 syndrome of inappropriate antidiuretic hormone secretion (SIADH). Length of stay was significantly different by groups; it was longest for pneumococcal meningitis (median: 16 days), and shortest for meningococcal meningitis (median: 11 days). Status at hospital discharge was available for all patients; six patients had neurological sequelae (7.1%). These included three children with hypoacusia, two requiring anticonvulsant therapy after a subdural hygroma, one with hydrocephalus. Patients with pneumococcal meningitis had a higher frequency of neurological sequelae (3/17; 17.6%), than all other patients (3/68; 4.4%), with a relative risk of 4.0 (95% CI: 0.9 -18.1; p = 0.057). No further factor or feature predictive of sequelae was identified. No deaths were observed.

**Table 3:** **Characteristics of in-hospital management by causative agent**

	***N. meningitidis***	***S. pneumoniae***	**Other**	**Not known**	**p-value**	**Total**
	**N. (29)**	**N. (17)**	**N. (10)**	**N. (29)**		**N. (85)**
Median length of stay days – (IQR)	11 (9 – 14)	16 (12 – 25)	15 (9 – 18)	13 (10 – 17)	0.006	13 (10 – 16)
Number of patients who underwent imaging – (%)	14 (48.3)	10 (58.8)	8 (80.0)	23 (79.3)	0.065	55 (64.7)
Number of patients with in-hospital audiological assessment – (%)	12 (41.4)	11 (64.7)	5 (50.0)	13 (44.8)	0.468	41 (48.2)
Number of patients with planned post-discharge audiological assessment – (%)	10 (35.7)	8 (50.0)	7 (70.0)	10 (34.5)	0.190	35 (42.2)
Number of patients with in-hospital complications – (%)	12 (41.4)	7 (41.2)	2 (20.0)	8 (27.6)	0.502	29 (34.1)
Number of patients with neurological sequelae at discharge – (%)	0 (0.0)	3 (17.7)	1 10.0)	2 (6.9)	0.094	6 (7.1)

IQR: interquartile range.

## Discussion

This study focused on clinical management of bacterial meningitis in children from a western Country, and was based on a series of cases prospectively collected in the post-vaccination era. We did not observe deaths; the rate of complications at discharge was 7.1%, which is lower than reported for the European Region by a systematic review of published papers from 1980 to 2008 on bacterial meningitis disabling sequelae [15], but higher than the rate of sequelae estimated in Greece from 1974 to 2005 [16].

Risk of complications and sequelae do vary by causative agent, being higher for meningitis due to *Streptococcus pneumoniae* than for other causative agents [15-18]. As expected on the basis of national vaccination coverage rates and epidemiological data, most of our cases were due to *N. meningitidis* and *S. pneumoniae. S. pneumoniae* cases were less frequent but more severe than other meningitis, with a 17.6% reported rate of neurological sequelae at discharge. The proportion of pneumococcal meningitis sequelae is consistent with results from meta-analysis, reporting a median of 24% (IQR: 14-40%).

Heptavalent pneumococcal conjugate vaccine (PCV7) has been available in Italy since 2001; the 13-valent pneumococcal conjugate vaccine (PCV13) has been introduced at the end of 2010, and it could prevent 94% of invasive infections due to *S. pneumoniae* [19]. Evaluation of vaccine effectiveness was beyond the objectives of this study, although in the future it will be interesting to review cases of meningitis before and after PCV13 introduction at national level. It should however be noted that in 2008, PCV7 coverage in Italy by 24 months of age was 55% on a national basis, with wide regional variations [4]; no more recent national coverage data are available. Improving pneumococcal vaccination coverage rates should be a priority at the National level, in order to prevent disabling meningitis cases as well as other invasive pneumococcal infections.

Current literature reports that complications and sequelae are less common in meningococcal meningitis [15,18], and our results confirm this finding. *N. meningitidis* caused however the majority of our cases, as well as most of meningitis cases reported at the national level [20]. In 2008, conjugate meningococcal C (MenC) vaccine coverage was even lower than observed for PCV7, being 37% by 24 months of age, and 16% among adolescents [4]. In 2011, approximately 50% of reported cases were due to serogroup B [20]. The availability of conjugate meningococcal B vaccine, licensed in the European Union in January 2013 but recommended in Italy since 2014 only in a minority of Regions and not at a national level, can guarantee high degree of protection against meningococcal meningitis [21]. As underlined for PCV13, achieving and maintaining optimal vaccination coverage for MenC and meningococcal B vaccine is crucial.

In Italy, primary care is provided to children by National Health Care pediatricians and hospital emergency access is free for all individuals. In our experience, approximately 80% of patients who were not transferred from other centers sought for hospital care advised by primary care physicians and 50% had received an antimicrobial treatment prior to hospital admission. Timely assistance and early treatment could have contributed to the favorable outcome that was observed in the majority of cases.

The choice of antibiotic is an issue to consider for acute bacterial meningitis management. First line recommended treatment of suspected meningitis is cefotaxime plus amoxicillin or ampicillin for infants <1-3 months, and ceftriaxone for infants and children ≥1-3 months [9]. Vancomycin plus a third-generation cephalosporin has been recommended as empirical treatment in Countries with prevalence of antimicrobial-resistant isolates (10), or in children who had prolonged exposure to antibiotics [9]. Italy is one of the European Countries with higher prevalence of antimicrobial-resistance; 12% of *S. pneumoniae* invasive isolates in 2012 were non susceptible to penicillin [22], and 5% of isolates in years 2006–2010 were resistant or intermediate to ceftriaxone according to the CLSI breakpoints for meningitis (MIC > 1 μg/μL) [23]. In our observation, a minority of *S. pneumoniae* cases were treated with a third-generation cephalosporin alone, while almost half of patients received an association of a third-generation cephalosporin with vancomycin or teicoplanin. We did not collect information on antimicrobial resistance, and we did not investigate how in vitro susceptibility testing results guided decision on choosing specific antimicrobial agent. However, the circulation of ceftriaxone resistant strains throughout the Country could justify the frequent use of third-generation cephalosporins associated with glycopeptides.

The rationale for use of corticosteroids in addition to antibiotics is their anti-inflammatory action. U.S. guidelines published in 2004 recommended adjunctive dexamethasone therapy in infants and children for Hib meningitis, because there were evidences supporting a clinical benefit when commenced with or before antibiotic therapy [10]. In 2010, UK National Collaborating Centre for Women’s and Children’s Health considered administration for infants or children > three months with suspected or confirmed bacterial meningitis, after interpretation of CSF parameters [9]. In 2013, a systematic review showed that corticosteroids reduced mortality in patients with meningitis due to *S. pneumoniae* and reduced severe hearing loss in children with meningitis due to Hib [24]; authors concluded that data support use in children in high-income countries.

In our results, dexamethasone was administered to almost all patients with pneumococcal meningitis (82%), and to the majority of patients with meningitis due to other agents or without known cause (59%). The study design does not allow to explore the possible association between corticosteroids and clinical outcome, but it is possible that administration had contributed to the low rate of complications we have observed. When administered, dexamethasone should be given within 1 hour of first antibiotic dose, and continued for no longer than 4 days [9]. Adherence to these recommendations was suboptimal, since timing of first dose was appropriate in 63.5% of total cases and median duration of administration was 6 days. Further studies should be conducted to describe determinants for appropriateness of corticosteroid adjunctive therapy and to define an evidence-based protocol on corticosteroid use in children with bacterial meningitis.

This study has a number of limitations. In Italy there are more than 450 pediatric units; centers who participated to this study represent a small proportion of all children’s hospitals and pediatric wards. The objective of the study was to evaluate clinical management of children hospitalized for acute bacterial meningitis, and we cannot infer conclusions on disease incidence and long-term outcome. Hospitals participated on a voluntary basis; a selection bias may have occurred due to participation of centers where compliance to recommendations for acute bacterial management is higher. We collected information on in-hospital treatment and patient status at discharge; the rate of sequelae at discharge may be underestimated because in-hospital audiological evaluation was not documented for all children. It is well known that neurological sequelae, such as hearing loss and cognitive difficulties, may be diagnosed post-discharge [15]. We had no long-term follow up data, but the low rate of patients for whom the audiological assessment was planned before discharging is in our experience a reason for concern.

## Conclusions

In a context of high accessibility to health services, we observed a rate of meningitis sequelae at discharge which is similar to what reported by other western countries. Most meningitis cases were due to *N. meningitidis* and *S. pneumoniae*. Reaching and maintaining adequate vaccination coverage against pneumococcal and meningococcal invasive infections remains a national priority to prevent bacterial meningitis cases.
